# Emerging Roles of Redox-Mediated Angiogenesis and Oxidative Stress in Dermatoses

**DOI:** 10.1155/2019/2304018

**Published:** 2019-04-16

**Authors:** Dehai Xian, Jing Song, Lingyu Yang, Xia Xiong, Rui Lai, Jianqiao Zhong

**Affiliations:** ^1^Department of Anatomy, Southwest Medical University, Luzhou 646000, China; ^2^Department of Dermatology, The Affiliated Hospital of Southwest Medical University, Luzhou 646000, China

## Abstract

Angiogenesis is the process of new vessel formation, which sprouts from preexisting vessels. This process is highly complex and primarily involves several key steps, including stimulation of endothelial cells by growth factors, degradation of the extracellular matrix by proteolytic enzymes, migration and proliferation of endothelial cells, and capillary tube formation. Currently, it is considered that multiple cytokines play a vital role in this process, which consist of proangiogenic factors (e.g., vascular endothelial growth factor, fibroblast growth factors, and angiopoietins) and antiangiogenic factors (e.g., endostatin, thrombospondin, and angiostatin). Angiogenesis is essential for most physiological events, such as body growth and development, tissue repair, and wound healing. However, uncontrolled neovascularization may contribute to angiogenic disorders. In physiological conditions, the above promoters and inhibitors function in a coordinated way to induce and sustain angiogenesis within a limited period of time. Conversely, the imbalance between proangiogenic and antiangiogenic factors could cause pathological angiogenesis and trigger several diseases. With insights into the molecular mechanisms of angiogenesis, increasing reports have shown that a close relationship exists between angiogenesis and oxidative stress (OS) in both physiological and pathological conditions. OS, an imbalance between prooxidant and antioxidant systems, is a cause and consequence of many vascular complains and serves as one of the biomarkers for these diseases. Furthermore, emerging evidence supports that OS and angiogenesis play vital roles in many dermatoses, such as psoriasis, atopic dermatitis, and skin tumor. This review summarizes recent findings on the role of OS as a trigger of angiogenesis in skin disorders, highlights newly identified mechanisms, and introduces the antiangiogenic and antioxidant therapeutic strategies.

## 1. Introduction

The complex process, regulated by proangiogenic and antiangiogenic factors, scientifically understood as the beginning formation of new blood vessels from existing ones, is known as angiogenesis [1]. New blood vessel formation, based on the balance of proangiogenic and antiangiogenic factors, is overwhelmingly responsible for most physiological events, such as embryogenesis, organ regeneration, body growth and development, skin renewal, and wound healing [2–4]. In the skin, angiogenesis is reactivated during skin renewal, wound healing, and tissue repair; furthermore, in these conditions, many angiogenic factors are released by activated keratinocytes and some inflammatory cells and jointly function to promote skin recovery and rejuvenation [5]; however, this process may be impaired by excessive angiogenic factors. In certain pathological conditions, these factors become overmuch and the balance between angiogenic promoters and inhibitors shifts, resulting in an angiogenic switch. The most well-known conditions where this switch is seen are malignant and inflammatory skin disorders as well as other pathological events, e.g., age-related macular degeneration, rheumatoid arthritis, tumor growth, proliferative retinopathies, and skin diseases (psoriasis, atopic dermatitis (AD), systemic sclerosis (SSc), cutaneous carcinoma, etc.) [5–8]. Either physiological or pathological angiogenesis is in need of initial mediation by various proangiogenic factors, consisting of endothelial growth factor (VEGF), fibroblast growth factors (FGF), interleukin-8 (IL-8), platelet-derived growth factor (PDGF), placental growth factor (PGF), angiopoietin-1 (Ang-1), and transforming growth factor-*β* (TGF-*β*) [9]. These proangiogenic factors subsequently induce a continuous recruitment of inflammatory cells to participate in the pathological process, which in turn serve as a substantial source of reactive oxygen species (ROS) [10, 11]. More importantly, excessive ROS trigger oxidative stress (OS), further promoting angiogenesis, damaging cells/tissue, and resulting in a variety of pathological changes [12].

**Figure 1:**
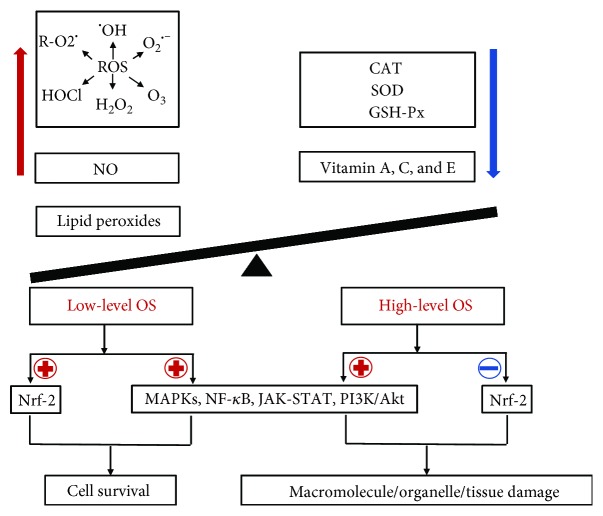
The process of oxidative stress (OS) generation. OS occurs when prooxidants (e.g., ROS, NO, and lipid peroxides) outbalance antioxidant defenses (e.g., SOD, CAT, and GSH-px). OS mediates in physiological or pathological events by activating/suppressing multiple redox signaling pathways (e.g., Nrf-2, MAPK, NF-*κ*B, PI3K/Akt, and JAK-STA). For example, high-level OS may induce the damage of macromolecules (DNA, proteins, and lipids), organelles (mitochondria and membranes), and even the whole tissues, whereas low-level OS may contribute to cell survival. In this process, ROS, including radical and nonradical ROS such as O_2_^•-^, ^•^OH, R^−^O_2_^•^, O3, HOCl, and H_2_O_2_, play a pivotal role in the generation of OS. ⊕ means “to promote or enhance”; ⊖ means “to inhibit or suppress.”

OS is frequently considered as an imbalance of redox originating from the overproduction of prooxidants (e.g., ROS, reactive nitrogen species (RNS), nitric oxide (NO), and lipid peroxides) or from the insufficiency of antioxidants/antioxidant enzymes, like superoxide dismutase (SOD), catalase (CAT), and glutathione peroxidase (GPx) (Figure 1) [13, 14]. Although the definition of OS is controversial, OS is currently regarded as a state in which stationary ROS/RNS transiently or gradually accumulate and ascend, further damaging cellular constituents and disturbing cellular metabolism [15]. Based on its intensity, OS is classified as basal OS (BOS), low-intensity OS (LOS), intermediate intensity OS (IOS), and high-intensity OS (HOS). OS, meanwhile, is categorized as mild OS (MOS), temperate OS (TOS), and severe OS (SOS) according to its degree [16]. In the process of OS, multiple redox signaling pathways are involved, primarily containing the mitogen-activated protein kinases/activator protein-1 (MAPK/AP-1), nuclear factor kappa-light-chain-enhancer of activated B cells (NF-*κ*B), Janus kinase-signal transducer and activator of transcription (JAK-STAT), nuclear factor erythroid 2-related factor (Nrf-2), phosphatidylinositol-3-kinase/protein kinase B (PI3K/Akt), and Toll-like receptor- (TLR-) mediated signal transduction pathway [17]. Through these signaling pathways, OS mediates in physiological or pathological events. For example, MOS contribute to cell survival, whereas SOS may damage macromolecules (DNA, proteins, and lipids) and organelles (mitochondria and membranes), even the whole tissues (Figure 1) [18]. ROS, the major contributors to OS, including oxygen-centered radical species (superoxide anion (O_2_^•-^), hydroxyl radical (^•^OH), and peroxyl radical (R^−^O_2_^•^)) and nonradical compounds (ozone (O_3_), hypochlorous acid (HOCl), and hydrogen peroxide (H_2_O_2_)), are often generated by various categories of cells like endothelial cells (ECs), perivascular adipocytes, epithelial cells, smooth muscle cells, and adventitial fibroblasts [17]. In a physiological context, ROS have important roles in cell/tissue physiological processes including cell signaling, homeostasis, skin regeneration/renewal, and wound healing. In the skin in particular, ROS are mainly responsible for cell damage in the ageing process. ROS in low concentrations, nevertheless, participate in a substantial number of physiological cell redox signaling pathways to maintain redox equilibrium [19]; more importantly, ROS generated from immune cells are potently available for host defense [20]. As germicides or an important player in cellular signaling, they are also vital to wound healing and skin repair, while high-level ROS create a redox imbalance in the skin further causing severe “oxidative stress,” eventually leading to DNA, cell, and tissue damage [21–27]. Several studies have demonstrated that in high concentration, H_2_O_2_ could induce endothelial injury; however, H_2_O_2_ in low concentration generally stimulates angiogenesis in wound healing and skin repair [28]. Accumulating evidence also supports that ROS as well ROS-mediated OS are involved in the process of physiological and pathological angiogenesis [29, 30] and closely implicated in the pathogenesis and exacerbation of angiogenesis-related diseases containing dermatoses, neurodegenerative disorders, cardiovascular diseases, and metabolic disorders [31–38]. In this review, we provide an overview of the current knowledge of the link between OS and angiogenesis and their roles in certain skin diseases as well as the emerging therapeutic strategies.

## 2. Role of OS in Angiogenesis

With further knowledge of angiogenesis, the pathogenesis of angiogenesis to some extent gradually becomes clear. It arrives at a consensus that ROS-mediated OS plays a crucial role in the development of angiogenesis. Moreover, two signal pathways of angiogenesis mediated by OS have been identified. One is the VEGF-dependent signaling pathway, while another is the VEGF-independent pathway [10, 39, 40].

### 2.1. Generation of ROS in Angiogenesis

At present, it is demonstrated that ROS-mediated redox signaling has a central role in angiogenesis. ROS act as a double-edged sword in the vasculature. In a physiological process, ROS work as an important component of signaling events and play an important role in cellular differentiation and maintenance of homeostasis [41]. However, overproduction of ROS (O^−2^ and H_2_O_2_) in turn contributes to neovascularization [21]. In this process, two endogenous ROS sources are mainly involved in the angiogenesis; one is nicotinamide adenine dinucleotide phosphate (NADPH) oxidase of the NOX family, and another is mitochondrial electron transport chain reactions [21, 31, 42, 43]. NADPH oxidase, a major source of ROS in ECs, generates O_2_^•-^ by transferring electrons from NADPH to oxygen. There are seven isoforms of NADPH oxidases expressed in mammals, namely, Nox1, Nox2 (previously gp91phox), Nox3, Nox4, Nox5, Duox1, and Duox2. This NADPH oxidase homologue consists of the following subunits: gp91phox (newly termed Nox2), p22phox, p40phox, p47phox, p67phox, and GTPase Rac1 [35, 44–46]. NADPH oxidase may be activated by diverse growth factors including VEGF, angiopoietin-1, ischemia, and hypoxia, and then, ROS derived from NADPH oxidase mediate in VEGFR-2 autophosphorylation [45, 47]. Apart from NADPH oxidase, ROS, the intracellular ROS in particular, were as well originated from the mitochondria. In the mitochondria, over 95% of oxygen consumed by cells affords water molecule production via redox reactions. But at complexes I and III in the transport chain, less than 4% of oxygen, which is reduced to superoxide anion instead of water, can generate OS [31, 45].

### 2.2. OS and Physiological Angiogenesis

Angiogenesis is physiologically essential for skin renewal, wound healing, tissue repair, skeletal remodeling, individual reproduction, etc. Among these physiological events, wound healing is a typical process involving angiogenesis and OS [20]. In this process, angiogenesis is induced by tissue hypoxia and ROS in either a VEGF-dependent way or a VEGF-independent way [48]. Low-concentration ROS facilitate angiogenesis in mouse wound healing and skin repair, which are involved in VEGF and its receptor signaling [49]. As the potent inducer of VEGF, hypoxia inducible factor 1 (HIF1) activated by ROS promotes angiogenesis via triggering VEGF expression during wound repair [50]. Thus, angiogenesis could be induced by ROS-mediated OS in a VEGF-dependent manner in wound healing. Increasing evidence has verified that VEGF can promote angiogenesis via binding to VEGF receptor-2 (VEGFR-2) in endothelial cells. The binding of VEGF to VEGFR-2 allows to activate a series of signal transduction molecules, including phospholipase C gamma (PLC*γ*) and phosphatidylinositol 3-kinase (PI3K), further stimulates the Raf-MAPK-ERK (mitogen-activated protein kinase/extracellular signal-regulated kinase) pathway, and finally facilitates angiogenesis [51, 52]. It is therefore considered that VEGF/VEGFR-2 signaling is a crucial signal transducer in both physiologic and pathologic angiogenesis. Apart from VEGF, other soluble factors like PDGF play an important role in angiogenesis during wound healing, which is dependent on H_2_O_2_ for its biological function [53]. Upon activation of the PDGF pathway, signaling occurs via the PI3K/Akt complex pathway and MAPK molecules [54]. Besides, endogenous 2-*ω*-carboxyethyl pyrrole (CEP), one major member of the carboxyalkyl pyrrole (CAP) family, is recognized by Toll-like receptor2 (TLR2) on endothelial cells and then activates MyD88-dependent signaling to promote angiogenesis at the wound site, which, in turn, accelerates wound healing. Consequently, OS also acts as the chief mediator of the VEGF-independent pathway in angiogenesis during the wound repair process [10].

### 2.3. OS and Pathological Angiogenesis

Pathological angiogenesis, fundamentally similar to physiological angiogenesis, is also affected by OS in VEGF-dependent and VEGF-independent ways, which proceeds in an unbalanced and uncontrolled fashion, finally resulting in an excessive and abnormal vascular pattern [55].

#### 2.3.1. VEGF-Dependent Signaling Pathway

As one of the major angiogenesis factors, VEGF stimulates EC proliferation and migration via binding to VEGFR-2 regardless of physiological status or pathological condition, further activates several downstream signaling cascades, such as mitogen-activated protein kinases (MAPKs), PI3k/AKT, or eNOS, and eventually leads to physiological or pathological angiogenesis. Physiological angiogenesis like wound angiogenesis has been discussed before, and the mechanism of pathological angiogenesis is as follow.

The VEGF signal is essential for homeostasis and vascular development, which is always influenced by ROS [35]. Increasing evidence has indicated that most OS-related angiogenesis depends on VEGF involvement. Xia et al. showed that NADPH oxidase-dependent ROS stimulated VEGF secretion and facilitated excessive angiogenesis in a tumor microenvironment through the HIF-1*α*-mediated VEGF pathway, further promoting tumor growth [56]. ROS from follicle-stimulating hormone (FSH) triggered HIF-1*α* signal and activated the VEGF signaling pathway by binding HIF-1*α* to the VEGF promoter and further accelerated excessive angiogenesis and finally contributed to ovarian epithelial cancer progression [57]. Likewise, products of oxidation exemplified by oxidized phospholipids (OxPLs) stimulate VEGF expression both *in vivo* and *in vitro*, thereby interacting with VEGFR-2 and triggering angiogenesis [58]. Especially, oxidized low-density lipoproteins (OxLDL) originated from OS could strongly induce HIF-1a and VEGF expression in monocyte macrophages and significantly enhance tube formation in cocultured endothelial cells [59–61]. As Toll-like receptor (TLR) ligands, poly (I:C) and lipopolysaccharide (LPS) both generated from OS, are also able to encourage angiogenesis via stimulating VEGF secretion or production and activate HIF-1*α* and the TLR pathway in a TLR-dependent manner [55, 62]. In addition, nitric oxide (NO) is considered to be one of the major contributors to angiogenesis and it has a capability of increasing the expression of HIF-1*α* and VEGF, thereby leading to angiogenesis [63]. Thus, ROS-promoting angiogenesis is dependent on VEGF and the HIF-1*α*/VEGF/VEGFR-2 pathway is a key molecular mechanism of OS-mediated angiogenesis [60].

#### 2.3.2. VEGF-Independent Signaling Pathway

Apart from the VEGF-dependent pathway, another novel mechanism of OS-mediated angiogenesis in a VEGF-independent manner recently has been demonstrated. Nowadays, because of some malignant tumors being resistant to anti-VEGF therapy, it is widely considered that the existence of VEGF-independent signaling is mainly responsible for this treatment-resistant event. In most cases, this resistance to anti-VEGF is linked with inflammation and infiltration of myeloid cells, which could create substantial oxygen tension and result in the accumulation of CEPs and finally accelerate neovascularization in a VEGF-independent manner [10]. There are two main VEGF-independent signaling pathways involved in angiogenesis, the CEP/TLR2/MyD88 axis and ROS/ataxia-telangiectasia mutated (ATM)/p38*α* pathways [10, 64]. The former mediates proangiogenesis and involves the accumulation of new lipid oxidation products, e.g., CAP protein adducts [55, 65]. CEP acts as a potential biomarker for OS-induced vascular disorders and has the same proangiogenic effect as VEGF *in vitro* [66]. It has been demonstrated that TLRs not only serve as guardians of innate immunity but also function as prominent contributors to angiogenesis [67]. At present, it has been discovered that several proangiogenic ligands of TLRs produced by OS promote angiogenesis in a VEGF-independent way, such as CEP (a TLR2 ligand), macrophage-activating lipopeptide-2 (MALP-2) (a TLR2/6 ligand), and LPS (a TLR4 ligand). The molecular pattern of CEP, for example, is recognized by TLR2 on endothelial cells and triggers the MyD88-dependent signal to accelerate neovascularization [68]. LPS could stimulate endothelial sprouting directly *in vitro* through a TRAF6-mediated activation of NF-*κ*B and JNK [69]. Angiogenesis is also induced via GM-CSF by TLR2/6 ligand binding to its receptor [70–72].

**Figure 2:**
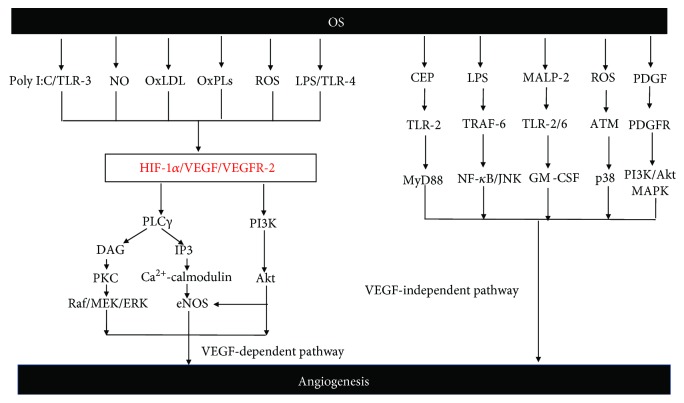
Schematic illustration of angiogenesis signaling pathways induced by OS. According to different responses to OS, two signal pathways of angiogenesis are covered, namely, the VEGF-dependent signaling pathway and VEGF-independent signaling pathway. In the VEGF-dependent pathway, ROS, NO, OxLDL, and OxPLs strongly stimulate the expression of HIF-1a and VEGF. Meanwhile, Poly I:C and LPS promote the expression of HIF-1*α* and VFGF by coupling with their specific receptors (TLR3 and TLR4). These further combine to the downstream receptor VEGFR-2 and facilitate angiogenesis by activating the HIF-1*α*/VFGF/VEGFR-2 signaling pathway in a VEGF-dependent manner. On the other hand, many mediators are involved in the VEGF-independent pathway, including CEP, LPS, MALP-2, PDGF, and ROS. CEP/yyMALP-2 initially couples to their receptors (TLR2/6), then sensitizes specific downstream targets (e.g., MyD88 and GM-CSF), and finally promotes angiogenesis. Meanwhile, ROS activate the P38 pathway via inducing the activation of ATM and ultimately result in angiogenesis. Besides, LPS is considered to induce angiogenesis through a TRAF6-mediated activation of NF-*κ*B and JNK. As another soluble mediator, PDGF triggers PI3K/Akt and MAPK signaling by binding to its receptor and promotes neovascularization.

On the other hand, the latter, namely, ATM kinase, known for its function in the regulation of cell cycle and DNA damage repair, has been identified as an alternative mediator of OS-induced angiogenesis [73–75]. Remarkably, compared to CEP-TLR2 in angiogenesis, ATM in angiogenesis is uniquely limited to promoting the pathological process and ATM activation enhances no other cells but endothelial cell proliferation, which provides a probability for anti-ATM therapy [73]. As the downstream of ATM in endothelial cells, p38 is also involved in response to ROS; diminishing of ATM also suppressed angiogenesis even in the absence of VEGF inhibitors, suggesting a VEGF-independent proangiogenic role of ATM [73].  Figure 2 sketches two pathways of OS-mediated angiogenesis.

## 3. OS and Angiogenesis in Dermatoses

Growing evidence supports that OS and angiogenesis are both closely implicated in the occurrence and development of some skin diseases, such as psoriasis, AD, malignant melanoma (MM), Behcet's disease (BD), and scleroderma. However, the specific mechanism still remains unclear; thus, we concentrate on recent findings to present the possible mechanism of OS and angiogenesis in these cutaneous diseases.

### 3.1. OS and Angiogenesis Associated with Psoriasis

Psoriasis is a common chronic inflammatory skin disease approximately affecting 2% of the population. It characteristically manifests as erythema and papules/plaques accompanied by thick silvery-white scales. Nowadays, there is a wide range of options available for the treatment of psoriasis, such as topical therapies, phototherapy, older small-molecule systemic agents (e.g., methotrexate, cyclosporine, acitretin, and fumaric acid), the newer oral phosphodiesterase-4 inhibitor apremilast, and the biologics (e.g., etanercept, adalimumab, infliximab, and ustekinumab) [76]. Despite that these therapies offer a certain efficacy, patients scarcely get satisfaction with substantial psoriatic lesion clearance, symptom relief, and improvements in quality of life [77]. Thus, there is a pressing need to develop some novel effective remedies. Although the etiology of psoriasis still remains unclear, it is thought that oxidative and angiogenic mechanisms both get involved in the pathological process of psoriasis. As one of major pathological features of psoriasis, angiogenesis has been persistently studied and various proangiogenic mediators have been identified in the psoriatic skin. Heidenreich et al. revealed a large spectrum of proangiogenic factors to mediate in psoriasis, including VEGF, HIF-1*α*, TNF, angiopoietins, IL-8, IL-17, and TGF-*α* [78].VEGF expression, in particular, remarkably elevated in the psoriatic serum and lesions. Moreover, ROS induced VEGF releasing from various cell types, whereas VEGF in turn promoted endothelial cell migration and proliferation through an increase of intracellular ROS. Thus, the VEGF pathway may be a crucial link between OS and angiogenesis in psoriasis, especially for the HIF-1*α*/VEGF signaling pathway playing a synergistic role in the neovascularization of psoriasis [79, 80]. By upregulating the expression of cell adhesion molecules, VEGF could enhance the migration of leukocytes into the psoriatic skin and increase oxygen consumption, further activating HIF-1*α* and perpetuating the angiogenic/inflammatory cycle of psoriasis [30, 81]. Furthermore, OxPLs afford the pathogenesis of psoriasis through enhancing VEGF generation from keratinocytes [30]. Besides, Elias et al. discovered that epidermal VEGF knockout mice scarcely appeared acanthosis after barrier disruption, suggesting an important contributor for VEGF to the development of keratinocyte hyperplasia. Hence, ROS-VEGF signaling may be a potential target for the treatment of psoriasis. However, the specific relationship between OS and angiogenesis in psoriasis requires to be further studied, which is conducive to fully clarify the pathogenesis of psoriasis and expand the optimal treatments for this disease.

### 3.2. OS and Angiogenesis Associated with AD

AD, a chronic inflammatory skin disease, adversely affects many people especially young children [82]. The current management of AD covers avoidance of triggering factors, skin care, and anti-inflammatory therapy (mostly topical corticosteroids and topical calcineurin inhibitors). Once these first-line approaches are unsuccessful, systemic therapy or phototherapy tends to be carried out as second-line treatment [83]. After being treated with these vehicles, most symptoms may be relieved. However, long-term use of the above therapeutic probably causes many side effects such as skin atrophy and dryness, photoaging, and potential occurrence of cutaneous malignancies [84]. Therefore, some novel therapies are needed for the management of AD. The pathogenesis of AD is complex and still poorly understood. Recently, emerging evidence suggests that OS is a potential key factor in the pathogenesis of AD [85]. OS is implicated in AD for several decades and remains present throughout the disease, including the onset of AD, the development of AD, and the exacerbation of AD. Moreover, excessive ROS overwhelm and destroy the skin antioxidant defense, which ultimately lead to AD progression and exacerbation [86]. Apart from OS, angiogenesis, as a hallmark of chronic inflammatory disorders, also gets involved in AD [87]. Several angiogenic factors contribute to the presence of angiogenic switch in the AD skin, such as VEGF, Angs, and IL-17. It has been demonstrated that angiogenesis is dysregulated in AD patients or models and high-level VEGF is detected in AD patient lesions. Meanwhile, Chen et al. discovered that a progressive increase of VEGF-A mRNA appeared in the skin of an AD mouse model [88]. Taken together, both OS and angiogenesis are mainly responsible for the pathogenesis of AD and the VEGF pathway may be a potential link between OS and angiogenesis in AD. Therefore, specific inhibitors targeting various mediators (e.g., VEGFs), receptors (e.g., VEGFRs and Tie-2), and oxides offer a promising foreground for the treatment of AD [7].

### 3.3. OS and Angiogenesis Associated with MM

MM, a malignant tumor of melanocytes, is accounted for about 10% of skin cancers, but it is responsible for over 90% of skin cancer deaths. For years, the cornerstones of cancer treatment have been surgery, chemotherapy, and radiation therapy. During the last decade, new strategies emerge from antitumor therapy for MM, including immunotherapy (e.g., checkpoint blockades) and targeted therapy (e.g., protein kinase inhibitors) or their combination [89]. Despite of extensive novel approaches serving for MM, the response rate is rarely higher than 20% and drug resistance is very common [90]. Up to date, rarely effective treatment has been approved for MM due to these reasons. As a result, it is urgent to invent other alternatives and targeted therapies [91, 92]. Compared to other tumors, MM is known for abundant ROS that exist in the primary tumor environment [93, 94]. ROS from OS at one time had been recognized as a powerful weapon for the immune system to kill tumor cells [95]. However, once MM cells successfully escape ROS-induced apoptosis, persistent ROS tend to favor melanoma survival, proliferation, and metastasis through activating several related pathways [96]. Thus, ROS and ROS-mediated OS are closely implicated in different stages of MM. Elevated ROS could trigger the occurrence of OS, which further disrupt the homeostasis of melanocytes, affect the epigenetic regulation, and induce gene mutation, ultimately leading to cancer generation [97]. Accordingly, much efforts have been made to battle melanoma by using antioxidants so far [98]. Moreover, ROS and ROS-mediated OS would promote MM angiogenesis in a VEGF-dependent or VEGF-independent manner; in their publication, Bald et al. as well have emphasized the importance of the vascular network for MM [99]. Several angiogenic factors (e.g., VEGF, bFGF, PIGF, PDGF, IL-8, and Ang-1) have been found to highly express in primary skin MM, and these mediators further promote MM angiogenesis and metastasis [100]. In addition, intratumoral hypoxia encourages the consequent expression of HIF-*α* transcription factors, in turn modulating VEGF and transcriptional product expression and mediating in cell growth, metabolism, and death [101, 102]. Meanwhile, preclinical studies indicate that the inhibitors targeting VEGF or VEGFR are effective in slowing the growth and metastasis of MM in murine models [103, 104]. Apart from VEGF, PDGF and its receptor PDGFR-*β* are responsible for MM angiogenesis. PDGF signaling is also implicated in angiogenesis in a VEGF-independent fashion. Therefore, OS and angiogenesis play vital roles in the development of MM; VEGF and PDGF signaling, moreover, may be the key link to OS and angiogenesis, which probably become the potential targets for the treatment of MM [105].

### 3.4. OS and Angiogenesis Associated with BD

BD, a chronic and recurrent vasculitis disease, is characterized by various clinical manifestations including skin lesions, oral/genital ulcer, ocular symptoms/lesions, joint signs, and organ involvements [106–108]. Glucocorticoids, azathioprine, cyclophosphamide, and cyclosporine A are currently the mainstay of treatments in vasculo-Behcet's disease, but long-term use of these drugs may induce some systemic adverse reactions [109]. Once immunosuppressive and corticosteroid therapies fail, biologic agents (e.g., infliximab, alemtuzumab, and adalimumab) can help for vascular lesions. However, high cost may be an obstacle to their widespread application [110]. Although BD etiology keeps being unknown, recently, growing evidence supports that elevated OS and insufficient antioxidant capacity are primarily involved in the pathogenesis of BD [111–113]. In the process of BD attack, ROS overproduction from OS may in turn accelerate OS aggression, then lead to tissue damage, and ultimately result in the pathological and clinical manifestations of BD. More importantly, we have demonstrated in our previous studies that there is an abnormal OS indeed existing in BD and a skewed redox balance remains present throughout this disease [114]. Apart from the OS-mediated mechanism, vascular endothelial activation is also considered to be a major one in BD [115–117]. Nowadays, it has been confirmed that several angiogenesis-promoting molecules (namely, angiogenic promoters) get involved in the pathogenesis of BD, including IL-8, matrix metalloproteinases, E-selectin, vascular endothelial-cadherin, and VEGF [118]. Among them, VEGF, the dominant factor controlling angiogenesis, was found to highly express in BD serum and elevated-level VEGF was proportional to BD activity [119–121]. VEGF, at the same time, plays an active role in the maintenance and growth of vascular endothelial cells. Kamoun et al. thought that high-level VEGF was closely associated with high concentration of NO from OS in BD [122]. Thus, OS and angiogenesis are crucial in BD pathogenesis and OS zealously mediates in the process of angiogenesis. However, further studies are needed to investigate the underlying mechanisms of OS-mediated angiogenesis in BD, in order to develop new therapeutic strategies for BD patients to suppress OS and angiogenesis.

### 3.5. OS and Angiogenesis Associated with Scleroderma

Scleroderma, also known as systemic sclerosis (SSc), is a chronic immune-mediated connective tissue disease involving the skin, blood vessels, systemic organs, lungs, kidney, and gastrointestinal tract in particular [91]. SSc consists of two clinical subsets: one is limited cutaneous SSc (lc-SSc) and another is diffuse cutaneous SSc (dc-SSc). Because skin sclerosis can cause joint contracture, disability, and poor quality of life, various systemic treatments (e.g., penicillamine, cyclophosphamide, methotrexate, azathioprine, mycophenolate mofetil, intravenous immunoglobulin, and tyrosine kinase inhibitors) have been applied to alleviate the symptoms. These treatments, however, may cause severe side effects and offer inconsistent efficacy [123]. Phototherapy, another approach used to relieve skin sclerosis, provides a local effect on the skin without systemic involvement, but it alone cannot completely reverse skin sclerosis and it is just used as an adjunctive therapy together with other antifibrotic treatments (i.e., corticosteroids and pentoxifylline**)** [124]. Recently, it is thought that OS plays an important part in promoting scleroderma development, though SSc pathogenesis remains obscure [125]. Murrell proposed that an abnormal generation of ROS should be responsible for most of the pathologic features of SSc [126]. For example, ROS could stimulate the production of profibrotic cytokines (including PDGF and TGF-*β*) and proinflammatory factors, accelerate the activation and proliferation of fibroblasts, promote the synthesis of type I collagen, and induce vascular dysfunction [127]. By targeting ROS-generating NADPH oxidase, fibroblast activation and experimental skin fibrosis are inhibited *in vitro* and *in* vivo [128]. On the other hand, several abnormalities in regulating angiogenic responses in scleroderma indicate that aberrant angiogenesis may be another important pathogenic factor of scleroderma [129]. Hummers et al. have found that high levels of angiogenic factors were measured in patients with scleroderma, e.g., VEGF, PDGF-BB, FGF2, and PlGF [130]. Meanwhile, increased-level VEGF and VEGFR have been discovered in the serum and skin samples from scleroderma patients [131–135]. Besides, HIF-1a is more prevalent in SSc patients than normal subjects [136]. Accordingly, the pathogenesis of scleroderma is closely associated with OS and abnormal angiogenesis but further studies focused on the link between OS and angiogenesis in SSc are still needed, which may lead to the development of a new way for scleroderma treatment.

### 3.6. OS and Angiogenesis Associated with Rosacea

Rosacea is a common chronic inflammatory dermatosis, clinically characterized by erythema of the central face, episodic flushing, papules, and pustules [137, 138]. Skin care and pharmacologic treatments are the pillars of effective management of rosacea. Apart from existing topical agents (sodium sulfacetamide, azelaic acid, metronidazole, and the alpha-adrenergic agonist brimonidine) and systemic medications (tetracyclines, beta-blockers and isotretinoin), new therapies including serine protease inhibitors and mast cell stabilizers may ameliorate rosacea symptoms [139]. However, some of these approaches have not been approved by the Food and Drug Administration. Though the exact pathogenesis of rosacea needs to be clarified, OS and oxidation of lipids are considered as crucial factors to trigger and aggravate the inflammatory processes of rosacea. Increased OS and decreased antioxidants are determined in systemic circulation of rosacea [140, 141]. OS, in addition, is complicated in vascular changes, inflammation, and oxidative tissue damage in rosacea [142]. Therefore, antioxidants may be a potential strategy for treating rosacea. As an essential process in chronic inflammatory dermatoses, angiogenesis also contributes to the development of rosacea [143–145]. Amal et al. reported that VEGF expression elevated in cutaneous lesions of rosacea and was consistent with vascular histological changes which clinically presented as erythema and telangiectasia [146]. VEGF, indeed, has an important impact on the angiogenesis process, responsible for telangiectasia and increased vascular permeability, leading to cutaneous inflammation and the presence of papules, pustules, and nodules in rosacea [147, 148]. Thus, attenuation of OS and VEGF may be relevant approaches for the therapy of rosacea. However, more research should be carried out to clarify the relationship of OS and angiogenesis and provide a novel therapeutic way for rosacea.

## 4. Therapeutic Implications

**Figure 3:**
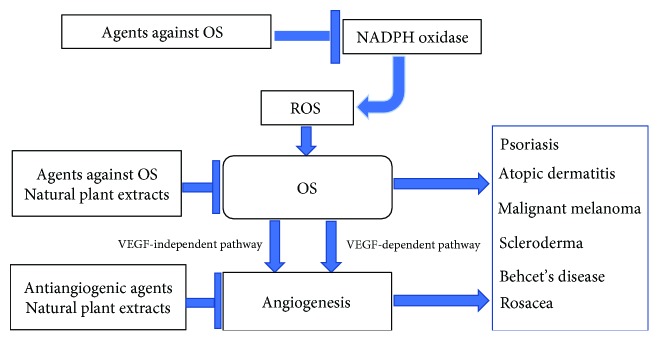
Strategies for dermatoses through mediating OS-induced angiogenesis and OS. Several novel therapies used for OS or angiogenesis-related skin disorders have been developed, including antiangiogenic agents, agents against OS, and natural plant extracts. These vehicles are potentially effective in management of dermatoses (e.g., psoriasis, AD, MM, scleroderma, BD, and rosacea) through mediating OS or angiogenesis-associated signal pathways.

Given that OS and OS-mediated angiogenesis have important roles in promoting various dermatoses, it should be fully suitable to develop novel therapies for skin disorders aimed at both aspects (Figure 3). As a major regulator of angiogenesis, VEGF and its pathway are considered as key targets for antiangiogenic therapy [149, 150]. Some effective drugs targeting VEGF have emerged from the pharmaceutical industry to inhibit new vessel formation.

### 4.1. Antiangiogenic Agents in the Management of Skin Diseases

Based on successful phase III trials, antiangiogenic therapeutics, anti-VEGF agents in particular (e.g., sorafenib, bevacizumab, and sunitinib), have entered the clinical practice in the USA and elsewhere. Strategies have been developed to inhibit the VEGF signaling pathway including anti-VEGF antibody therapy (e.g., bevacizumab), anti-VEGFR antibody therapy (e.g., ramucirumab), inhibitors of VEGFR-2 tyrosine kinases (e.g., apatinib), and inhibitors of angiogenic receptor tyrosine kinases (e.g., sunitinib, pazopanib, sorafenib, and regorafenib) [151]. Due to their antiangiogenic, antioxidative, and antiproliferative effects, phytochemicals are beneficial in the battle against cutaneous carcinoma [152]. Intraperitoneal injection of recombinant thrombospondin type 1 repeat domain (rTSR1) or a disintegrin-like and metalloproteinase with thrombospondin motifs 5 (ADAMTS5) could potently inhibit subcutaneous melanoma growth by diminishing angiogenesis, promoting apoptosis, and decreasing cell proliferation in the tumor tissue [153]. Antiangiogenic agent AE-941 from extracts of cartilage potentially provides a beneficial effect to treat cutaneous and systemic diseases especially for psoriasis [154]. It is speculated that cannabinoids have a potential role in treatment of psoriasis by controlling angiogenesis and inflammation through decreasing HIF-1*α* and VEGF levels [155]. Meanwhile, Kuang et al. also demonstrated that topical sunitinib ointment contributed to attenuate imiquimod-induced psoriasis-like inflammation through regulating the proliferation and apoptosis of keratinocytes via suppressing p-Stat3 and VEGF expression [156]. Besides, thalidomide effectively works in skin disorders such as BD through inhibition of VEGF- and FGF-2-mediated angiogenesis [157].

### 4.2. Agents against OS in the Management of Dermatoses

On the other side, it is quite beneficial to skin disorder recovery by employing OS-targeted drugs like antioxidants. Because OS-dependent angiogenesis is an important contributor to the progression of cancers, antioxidants may overcome the limitations of anti-VEGF therapy, especially in relation to tumor resistance. Related documents revealed that glabridin ameliorated imiquimod-induced psoriasis-like inflammation on BALB/c mice skin through improvement of antioxidant status and downregulation of proinflammatory cytokines [158]. By decreasing lipid peroxidation and modulating Ca^2+^ release, colchicine significantly induced protective effects on OS in the neutrophils of BD patients [159]. More importantly, high-dose vitamin C could effectively work in the skin diseases of MM and AD owing to its antioxidant protection [160]. Apart from the traditional antioxidants, NADPH oxidase, a key enzyme generation of ROS in neovascularization, potentially becomes the important target of pharmacological inhibitors. And NOX inhibitors are the most promising therapeutic option for diseases associated with OS. Among them, traditional NADPH oxidase inhibitors, such as apocynin and diphenylene iodonium, have no specificity and little isoform selectivity. Instead, several novel NOX inhibitors (GKT137831, ML171, and VAS2870) exhibit improved specificity for NADPH oxidases and NOX isoform selectivity [161].

### 4.3. Natural Plant Extracts in the Management of Dermatoses

Nowadays, natural extracts from plants increasingly arrest the attention from medical fields and pharmaceutical industry. Numerous natural extracts, like tea polyphenol, proanthocyanidins, and allicin, are potently beneficial to various skin disorders. As the main active ingredient of tea polyphenol, epigallocatechin-3-gallate (EGCG) could prevent OS-induced damage and suppress angiogenesis to avail against skin cancer and psoriasis basing on its antioxidant, antitumor, and antiangiogenic properties [162, 163]. Due to their powerful antioxidation, antiangiogenesis, antiproliferation, and antioncogenesis, proanthocyanidins have a wide utilization in the management of various OS-related and angiogenic complaints [164, 165]. Phenolic metabolites [166]. Moreover, we have proposed in our previous publications that proanthocyanidins are good for the treatment of psoriasis, AD, allergic purpura, SSc, rosacea, skin cancer, and other dermatoses [167]. Besides, our recent finding reveals that allicin, an active substance from garlic, has a favorable efficacy on BD by attenuation of OS and balance of oxidant/antioxidant status [168].

## 5. Conclusion

In summary, there are two main mechanisms implicated in the area bridging angiogenesis and OS; one is the VEGF-dependent signaling pathway (HIF/VEGF signaling), while another is the VEGF-independent signaling pathway (CEP/TLR2/MyD88 axis and ROS/ATM/p38*α* pathway). It is clear that OS and OS-derived angiogenesis are important contributors to the progression of chronic diseases and tumors. There is no doubt that both OS and angiogenesis participate in the development of certain skin diseases; however, a deeper understanding of the mechanisms behind OS and OS-dependent angiogenesis is necessary. There is a need for an investigation of multifaceted pathways involved in OS-induced angiogenesis in dermatoses and a specific target discriminating pathological vasculature from the physiological one. Therefore, in addition to the anti-VEGF drugs and OS inhibitors or antioxidants, it is necessary to develop some newly specific target strategies.
